# A Hybrid Algorithm of LSTM and Factor Graph for Improving Combined GNSS/INS Positioning Accuracy during GNSS Interruptions

**DOI:** 10.3390/s24175605

**Published:** 2024-08-29

**Authors:** Fuchao Liu, Hailin Zhao, Wenjue Chen

**Affiliations:** 1 School of Automation, Beijing Information Science & Technology University, Beijing 100192, China; 2 Beijing Key Laboratory of High Dynamic Navigation Technology, Beijing Information Science & Technology University, Beijing 100192, China

**Keywords:** long short-term memory (LSTM), combined GNSS/INS positioning, GNSS interruptions, factor graph

## Abstract

In urban road environments, global navigation satellite system (GNSS) signals may be interrupted due to occlusion by buildings and obstacles, resulting in reduced accuracy and discontinuity of combined GNSS/inertial navigation system (INS) positioning. Improving the accuracy and robustness of combined GNSS/INS positioning systems for land vehicles in the presence of GNSS interruptions is a challenging task. The main objective of this paper is to develop a method for predicting GNSS information during GNSS outages based on a long short-term memory (LSTM) neural network to assist in factor graph-based combined GNSS/INS localization, which can provide a reliable combined localization solution during GNSS signal outages. In an environment with good GNSS signals, a factor graph fusion algorithm is used for data fusion of the combined positioning system, and an LSTM neural network prediction model is trained, and model parameters are determined using the INS velocity, inertial measurement unit (IMU) output, and GNSS position incremental data. In an environment with interrupted GNSS signals, the LSTM model is used to predict the GNSS positional increments and generate the pseudo-GNSS information and the solved results of INS for combined localization. In order to verify the performance and effectiveness of the proposed method, we conducted real-world road test experiments on land vehicles installed with GNSS receivers and inertial sensors. The experimental results show that, compared with the traditional combined GNSS/INS factor graph localization method, the proposed method can provide more accurate and robust localization results even in environments with frequent GNSS signal loss.

## 1. Introduction

With the rapid development of unmanned and mobile positioning, the demand for high-precision and high-robustness localization techniques is increasing [1]. Among modern navigation and positioning systems, the global navigation satellite system (GNSS) is widely used on a variety of occasions due to its global coverage and high-precision positioning capability [2]. However, in complex urban road environments such as tunnels, canyons, tall buildings, overpasses, or other situations where satellite signals are obstructed, GNSS systems are challenged with severe signal attenuation or complete loss of lock [3]. Such signal loss is a serious challenge for unmanned vehicles that rely on the GNSS for precise navigation and positioning, as well as intelligent transportation systems and other positioning systems that require high reliability and accuracy. In order to solve the problem of GNSS loss of lock, researchers have proposed a combined positioning method using the inertial navigation system (INS) and the GNSS [4,5]. The INS can provide relatively accurate position and velocity in a short period of time, but the acceleration and angular velocity information output from the inertial measurement unit needs to be integrated, and the error will keep accumulating with the increase of time, and it cannot provide high-precision positioning information independently for a long time. Therefore, by combining the INS with the GNSS [6,7], the global positioning information provided by the GNSS can be used to correct the accumulated errors of an INS and achieve more accurate and robust navigation and positioning. 

In the field of GNSS/INS integrated positioning, the Kalman filter (KF) and its derivative algorithms, such as the extended Kalman filter (EKF) [8,9] and the unscented Kalman filter (UKF) [10,11], have long been the mainstream techniques for handling state estimation of both linear and nonlinear systems. By fusing the global positioning of the GNSS and the autonomous positioning of the INS, these filtering methods have successfully improved the positioning accuracy and system robustness in a variety of environments. However, as the complexity of application scenarios increases, KF and its variants exhibit limitations in dealing with highly nonlinear problems, error accumulation, and occasions where global optimization solutions are required [12,13]. Especially in urban canyon or tunnel environments, where GNSS signals are susceptible to interference or occlusion, the problem of error accumulation of these algorithms under prolonged operation is particularly significant. To overcome these challenges, factor graphs are proposed as a new solution [14,15,16,17,18]. By constructing a global optimization framework that allows for a comprehensive consideration of the entire system state, rather than relying solely on sequential state updates, factor graphs exhibit higher accuracy and stability when dealing with nonlinear systems and performing long-term estimation. Compared to Kalman filtering and its variants, factor graphs not only handle highly nonlinear problems more naturally but also reduce the effect of error accumulation through global optimization, which significantly improves the performance of positioning systems in complex environments. The authors in [19,20] compared the combined navigation based on factor graphs and the EKF, and the factor graphs make the navigation and localization performance better than the EKF by constructing constraints with more historical data and multiple iterations. The authors in [21] compared the multi-source information fusion by using factor graphs and compared it with the federated filtering method. The localization error, stability, and data fusion time of the factor graphs is better than that of the federated filtering. The factor graph method shows a wide range of application prospects and advantages in the field of combinatorial localization [22,23,24], which opens up a new way to improve localization accuracy and system robustness.

In recent years, with the rapid development of deep learning technology, neural network-based methods have provided new perspectives for solving complex navigation and positioning problems [25,26,27]. A variety of neural network-based theoretical models and algorithms have been introduced in combined positioning and information fusion [28] to improve the continuity of the combined positioning system and the positioning accuracy in the GNSS out-of-lock environment. The authors in [29,30] used a multilayer perceptron (MLP) to assist in combined GNSS/INS localization and utilized the velocity, angular rate, and specificity information of the INS to predict pseudo-GNSS information in the event of GNSS interruption. However, when the model complexity is too high or the amount of training data is insufficient, the MLP is prone to overfitting the training data and falling into local optimization. The authors in [31] utilized the recurrent neural network (RNN) assisted localization to predict the current error when the GNSS is unavailable by inputting the amount of change in velocity and attitude with respect to the position and velocity error, which is used to compensate and correct the INS error. However, it still suffers from overfitting risk, gradient vanishing, or explosion problems. While long short-term memory (LSTM) networks can solve the common gradient vanishing problem of the RNN in long sequence learning, LSTM is able to deal with the time dependence of data compared to an MLP. The previously mentioned scholars have used various neural networks combined with Kalman and its variants to solve the combinatorial localization problem in the case of GNSS interruptions, whereas combinatorial localization methods using neural networks combined with factor graphs are less common.

In summary, to achieve robust and continuous localization in the absence of the GNSS, this paper proposes a combined localization method using an LSTM-assisted GNSS/INS pre-integrated factor graph in scenarios where GNSS signals are interrupted. This method first utilizes the pre-integrated factor graph algorithm to fuse GNSS and INS data, enabling high-precision positioning in environments with good GNSS signal availability. In the event of a GNSS lockout, the method uses a pre-trained LSTM model to predict the GNSS position increments, which are then used to estimate the position information during the GNSS outage. This prediction effectively compensates for the absence of GNSS position information during interruptions, ensuring the continuity and robustness of the localization system. The main contributions of this paper are as follows:

The paper describes an integrated GNSS/INS positioning framework based on factor graphs. It involves pre-integrating the IMU to reduce error propagation and enhance the overall accuracy of the factor graph-based combined positioning system. 

By training an LSTM neural network prediction model, GNSS position increments are predicted in the event of GNSS interruptions, generating GNSS information that is integrated with INS positioning results to improve the accuracy and robustness of the GNSS/INS combined positioning system.

The remainder of the paper is organized as follows: Section 2 introduces the factor graph theory, IMU pre-integration factors, GNSS factors, and the factor graph combined positioning model. Section 3 details the training and prediction methods of the proposed LSTM neural network prediction model. Road tests and result analysis are conducted in Section 4, and conclusions are presented in Section 5.

## 2. Information Fusion Methods Based on Factor Graphs

A factor graph is a graphical structure used to represent probabilistic graphical models, which illustrates the decomposition of multivariable functions into a product of local functions. Factor graphs are composed of variable nodes and factor nodes connected by undirected edges. Variable nodes represent system states, while factor nodes represent constraints or measurements between variables. In navigation positioning systems, the optimal navigation solution corresponds to the maximum a posteriori estimate of the system state. According to Bayesian theory, the posterior probability can be expressed as the product of a series of probability models [32].

(1) $$\begin{array}{c} P\left(x_{k}|z_{k}\right)=\frac{P(z_{k}|x_{k})P(x_{k}|x_{k-1})}{P(z_{k})}P(x_{k-1}|z_{k-1})\\ \;\;=\prod_{i=1}^{k}\frac{P(z_{i}|x_{i})P(x_{i}|x_{i-1})}{P(z_{i})}P(x_{0}) \end{array}$$

where $x_{k}$ represents the system state variable at time k, $z_{k}$ is the measurement value from the sensor at time k, $P(x_{0})$ is the likelihood probability density of the initial state variables, $P(x_{k}|z_{k})$ is the posterior probability density of the measurements, and $P(z_{k})$ is the probability density of the measurements at time k. Since the initial state is known and each positioning measurement is independent, the posterior probability is the product of the probability densities of state transitions and measurement predictions at each moment. The global conditional probability density function is proportional to the likelihood probability density and the state transition prior probability in the numerator mentioned above.

(2) $$\frac{P(z_{i}|x_{i})P(x_{i}|x_{i-1})}{P(z_{i})}P(x_{0})\propto P(z_{i}|x_{i})P(x_{i}|x_{i-1})$$

According to the maximum a posteriori probability criterion, the optimal estimation of the state variable can be represented by the maximum a posteriori probability density:

(3) $${\hat{X}}_{i}^{map}=\arg\max P(x_{i}|z_{i})$$

According to the principles of factor graphs, each probability density corresponds to a factor node within the graph. Thus, the above expression can be rewritten as follows:

(4) $${\hat{X}}_{i}^{map}=\underset{X}{\arg\max}\prod_{i}f_{i}(X_{i})$$

where the factor node $f_{i}(X_{i})$ can be represented as follows:

(5) $$f_{i}(X_{i})=\exp(-\frac{1}{2}{\left\|\left(h_{i}(X_{i})-Z_{i}\right)\right\|}_{\sum_{i}}^{2})$$

(6) $$r(x_{i},z_{i})=h_{i}(X_{i})-Z_{i}$$

where $h_{i}(X_{i})$ is the observation value, $\sum_{i}$ is the noise covariance matrix of the corresponding sensor, and $r(x_{i},z_{i})$ is the sensor measurement residual. For nonlinear optimization within the factor graph, the maximum a posteriori can be transformed into minimizing the sum of the nonlinear least squares [33], expressed as follows:

(7) $$\begin{array}{l} X^{map}=\underset{X}{\arg\min}\left\{-\log\prod_{i}f_{i}(X_{i})\right\}\\ \;\;=\underset{X}{\arg\min}\left\{\sum_{i}{\left\|r(x_{i},z_{i})\right\|}_{\sum_{i}}^{2}\right\} \end{array}$$

By combining the sensor factors derived from observations and prior information, the solution for maximum a posteriori probability is found, thus enabling the fusion of data from different sensors. Therefore, for the GNSS/INS integrated positioning factor graph model, the optimal estimate of the system state X can be represented as follows:

(8) $$X^{map}=\underset{X}{\arg\min}\left\{\begin{array}{l} \sum_{k}^{n}{\left\|r_{imu}(z_{b(k)}^{b(k-1)},X)\right\|}_{\sum_{k-1,k}^{imu}}^{2}\\ +\sum_{i}^{n}{\left\|r_{gnss}(z_{i}^{gnss},X)\right\|}_{\sum_{i}^{gnss}}^{2}\\ +{\left\|r_{p}-H_{p}X\right\|}_{\sum_{p}}^{2} \end{array}\right\}$$

where $\sum^{imu},\sum^{gnss}$ are the covariance matrices for the IMU preintegration and GNSS, respectively, and $r_{imu},r_{gnss}$ are the residuals of the IMU preintegration factors and GNSS positioning factors, which are further derived and explained later in the text. $\left\{r_{p},H_{P}\right\}$ is the prior information and $\sum_{p}$ is the prior covariance matrix. The prior factor is used in navigation positioning problems to introduce prior information or measurement information about the state variables, helping the positioning system to estimate the state more accurately.

### 2.1. IMU Pre-Integration Factor Node

**Figure 1:**
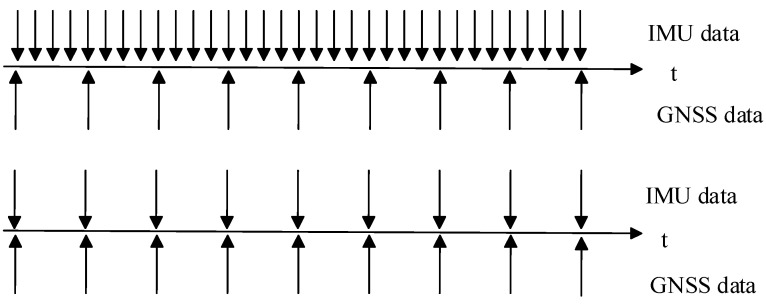
Pre-integration schematic diagram.

Typically, IMUs output data at a high frequency, and directly processing these high-frequency data can lead to a substantial computational burden. Raw IMU data consists of angular velocity and acceleration. Integration of angular velocity results in orientation, while acceleration is integrated to obtain velocity and further integration of velocity results in displacement. In this process, errors accumulate progressively. To address this issue, pre-integration is employed to preprocess IMU data [34]. Through pre-integration, high-frequency IMU data can be compressed into a single update over a period, reducing the error accumulation caused by multiple integrations. Essentially, the raw IMU data are integrated to compute the carrier’s relative displacement and rotational changes over a period. To ensure data synchronization and alignment, the IMU preintegration time interval is kept consistent with the GNSS sampling interval, and an IMU preintegration factor is added within each GNSS sliding window. The schematic diagram of IMU preintegration is shown in Figure 1.

In the integrated positioning system, the acceleration ${\tilde{f}}^{b}$ and angular velocity ${\tilde{\omega }}_{ib}^{b}$ measured by the IMU reflect the dynamic changes of the carrier [35]. The IMU measurement model can be represented as follows:

(9) $${\tilde{\omega }}_{ib}^{b}=\omega_{ib}^{b}+b_{\omega }+\varepsilon_{\omega },\;{\tilde{f}}^{b}=f^{b}+b_{a}+\varepsilon_{a}$$

where $\omega_{ib}^{b},f^{b}$ are the measured true values for the gyroscope and accelerometer, $b_{\omega },b_{a}$ are the biases for the gyroscope and accelerometer, and $\varepsilon_{\omega },\varepsilon_{a}$ are the noises for the gyroscope and accelerometer. According to the kinematic model of the INS, the differential equations for position, velocity, and attitude with respect to time can be obtained as follows:

(10) $$\left\{\begin{array}{c} {\dot{p}}_{b}^{n}=v_{b}^{n}\\ {\dot{v}}_{b}^{n}=C_{b}^{n}({\tilde{f}}^{b}-b_{a})+g^{n}\\ {\dot{q}}_{b}^{n}=\frac{1}{2}q_{b}^{n}⊗\left[\begin{array}{l} 0\\ \omega_{ib}^{b} \end{array}\right] \end{array}\right.$$

where n represents the navigation coordinate system (East-North-Up), b denotes the body coordinate system (Right-Front-Up), $p_{b}^{n}$ is the position in the n-frame, $v_{b}^{n}$ is the velocity in the n-frame, $q_{b}^{n}$ is the quaternion from the b-frame to the n-frame, $C_{b}^{n}$ is the attitude rotation matrix from the b-frame to the n-frame, and $g^{n}$ is the local earth gravity in the n-frame. By integrating the differential equations for position, velocity, and attitude over the time interval $\left[t_{k-1},t_{k}\right]$, the kinematic equations of the INS are obtained as follows:

(11) $$\left\{\begin{array}{l} p_{b(k)}^{n}=p_{b(k-1)}^{n}+\int_{t_{k-1}}^{t_{k}}v_{b(t)}^{n}dt\\ v_{b(k)}^{n}=v_{b(k-1)}^{n}+\int_{t_{k-1}}^{t_{k}}(C_{b(t)}^{n(t)}({\tilde{f}}^{b}-b_{a})+g^{n})dt\\ q_{b(k)}^{n}=q_{b(k-1)}^{n}⊗q_{b(k-1)}^{n(k-1)}⊗q_{b(k)}^{n(k-1)} \end{array}\right.$$

The IMU pre-integration from time $t_{k-1}$ to $t_{k}$ can be expressed as follows:

(12) $$\left\{\begin{array}{l} △p_{b(k)}^{b(k-1)}=\int \int_{t_{k-1}}^{t_{k}}C_{b(t)}^{b(k-1)}({\tilde{f}}^{b}-b_{a})dt^{2}\\ △v_{b(k)}^{b(k-1)}=\int_{t_{k-1}}^{t_{k}}C_{b(t)}^{b(k-1)}({\tilde{f}}^{b}-b_{a})dt\\ q_{b(k)}^{b(k-1)}={\left({\left(q_{b(k)}^{n(k)}\right)}^{-1}⊗q_{n(k-1)}^{n(k)}⊗q_{b(k-1)}^{n(k-1)}\right)}^{-1} \end{array}\right.$$

where $△p_{b(k)}^{b(k-1)},△v_{b(k)}^{b(k-1)},q_{b(k)}^{b(k-1)}$ represents the position pre-integration, velocity pre-integration, and attitude pre-integration. In factor graph optimization, a noise covariance matrix is required to weight the IMU factor, with the error state vector defined as follows:

(13) $$\delta z_{t}={\left[\begin{array}{ccccc} \delta p_{b(k)}^{b(k-1)} & \delta v_{b(k)}^{b(k-1)} & \delta q_{b(k)}^{b(k-1)} & \delta b_{\omega } & \delta b_{a} \end{array}\right]}^{T}$$

where $\delta p_{b(k)}^{b(k-1)},\delta v_{b(k)}^{b(k-1)},\delta q_{b(k)}^{b(k-1)}$ represent the pre-integrated measurement errors for position, velocity, and attitude, respectively, and $\delta b_{\omega },\delta b_{a}$ are the bias errors for the gyroscope and accelerometer. The continuous-time dynamics model for pre-integrated error states is represented as follows:

(14) $$\delta {\dot{z}}_{t}=F_{t}\delta z_{t}+G_{t}\varepsilon_{t}$$

where the state transition matrix $F_{t}$, the process noise matrix $G_{t}$, and the noise vector $\varepsilon_{t}$ are respectively represented as follows:

(15) $$F_{t}=\left[\begin{array}{ccccc} 0 & I & 0 & 0 & 0\\ 0 & 0 & -{\hat{C}}_{b(t)}^{b(k-1)}({\hat{f}}^{b}-b_{a})\times & 0 & {\hat{C}}_{b(t)}^{b(k-1)}\\ 0 & 0 & -({\hat{\omega }}_{ib}^{i}-b_{\omega })\times & -I & 0\\ 0 & 0 & 0 & 0 & 0\\ 0 & 0 & 0 & 0 & 0 \end{array}\right]$$

(16) $$G_{t}=\left[\begin{array}{cc} 0 & 0\\ 0 & {\hat{C}}_{b(t)}^{b(k-1)}\\ -I & 0\\ 0 & 0\\ 0 & 0 \end{array}\right],\;\varepsilon_{t}=\left[\begin{array}{c} \varepsilon_{\omega }\\ \varepsilon_{a} \end{array}\right]$$

The pre-integration covariance matrix can be expressed as follows:

(17) $$\sum_{b(t)}^{imu}=\Phi_{t}\sum_{b(t-1)}^{imu}{\Phi_{t}}^{Τ}+\int_{t-1}^{t}\Phi_{t}G_{t}Q{G_{t}}^{Τ}{\Phi_{t}}^{Τ}dt$$

where the initial covariance matrices $\sum_{b(t-1)}^{imu}=0$, $\Phi_{t}\approx Ι+F_{t}△t$, $Q=diag\left[\sigma_{\omega }^{2},\sigma_{a}^{2}\right]$.

The first-order Jacobian propagation equation can be expressed as follows:

(18) $$J_{k-1,t}=\Phi_{t}J_{k-1,t-1}$$

where the initial Jacobian matrix $J_{k-1,t-1}$ is the identity matrix. Based on the Jacobian and covariance matrices, a first-order expansion can be used to update the pre-integrated measurements of Equation (11), and the bias-updated pre-integrated measurements are calculated as follows:

(19) $$\left\{\begin{array}{l} △{\hat{p}}_{b(k)}^{b(k-1)}\approx △p_{b(k)}^{b(k-1)}+J_{b\omega }^{p}\delta b_{\omega }+J_{ba}^{p}\delta b_{a}\\ △{\hat{v}}_{b(k)}^{b(k-1)}\approx △v_{b(k)}^{b(k-1)}+J_{b\omega }^{v}\delta b_{\omega }+J_{ba}^{v}\delta b_{a}\\ {\hat{q}}_{b(k)}^{b(k-1)}\approx q_{b(k)}^{b(k-1)}⊗\left[\begin{array}{c} 1\\ \frac{1}{2}J_{b\omega }^{\phi }\delta b_{\omega } \end{array}\right] \end{array}\right.$$

where $J_{b\omega }^{p}$ is a submatrix of $J_{k-1,k}$, located according to $\delta p_{b(k)}^{b(k-1)}/\delta b_{\omega }$. This definition also applies to $J_{ba}^{p},J_{b\omega }^{v},J_{ba}^{v},J_{b\omega }^{\phi }$. Hence, the residual of the IMU pre-integration factor can be represented as follows:

(20) $$r_{imu}(z_{b(k)}^{b(k-1)},X)=\left[\begin{array}{c} △p_{b(k)}^{b(k-1)}-△{\hat{p}}_{b(k)}^{b(k-1)}\\ △v_{b(k)}^{b(k-1)}-△{\hat{v}}_{b(k)}^{b(k-1)}\\ 2{\left(q_{b(k)}^{n(k)}\right)}^{-1}⊗q_{n(k-1)}^{n(k)}⊗q_{b(k-1)}^{n(k-1)}⊗{\hat{q}}_{b(k)}^{b(k-1)}\\ b_{\omega (k)}-b_{\omega (k-1)}\\ b_{a(k)}-b_{a(k-1)} \end{array}\right]$$

### 2.2. GNSS Factor Nodes

The GNSS is highly valuable in open and unobstructed environments for providing absolute and long-duration positional information. However, in environments with obstructions such as urban high-rises, tunnels, and overpasses, GNSS signals may be blocked or interfered with. Thus, it becomes necessary to fuse data from other sensors to enhance navigation accuracy and robustness. The raw GNSS pseudorange and carrier phase observations can be expressed as follows:

(21) $$\left\{\begin{array}{l} p_{r}^{s}=\rho_{r}^{s}+t_{r}-t^{s}+I_{r}^{s}+T_{r}^{s}+b_{r}-b^{s}+\varepsilon_{p}\\ L_{r}^{s}=\rho_{r}^{s}+t_{r}-t^{s}+I_{r}^{s}+T_{r}^{s}+\lambda (N_{r}^{s}+B_{r}-B^{s})+\varepsilon_{L} \end{array}\right.$$

where $p_{r}^{s},L_{r}^{s}$ represents the pseudorange and carrier phase observations from receiver ‘*r*’ to satellite ‘*s*’; $\rho_{r}^{s}$ denotes the geometric distance from the satellite antenna phase center to the receiver antenna phase center; $t_{r},t^{s}$ refers to the receiver and satellite clock biases; $I_{r}^{s},T_{r}^{s}$ are the ionospheric and tropospheric delays; λ is the wavelength; $N_{r}^{s}$ is the carrier phase integer ambiguity; $b_{r},b^{s}$ are the pseudorange hardware delays at the receiver and satellite ends; $B_{r},B^{s}$ are the phase delays at the receiver and satellite ends; and $\varepsilon_{p},\varepsilon_{L}$ includes residual errors on the pseudorange and carrier phase observations, encompassing the sum of observation noise and multipath errors.

To enhance GNSS positioning accuracy and interference resistance, a dual-frequency ionosphere-free (*IF*) combination is used to eliminate the first-order ionospheric delay:

(22) $$\left\{\begin{array}{l} p_{r,IF(i,j)}^{s}=\frac{f_{i}^{2}}{f_{i}^{2}-f_{j}^{2}}p_{r,i}^{s}-\frac{f_{i}^{2}}{f_{i}^{2}-f_{j}^{2}}p_{r,j}^{s}\\ L_{r,IF(i,j)}^{s}=\frac{f_{i}^{2}}{f_{i}^{2}-f_{j}^{2}}L_{r,i}^{s}-\frac{f_{i}^{2}}{f_{i}^{2}-f_{j}^{2}}L_{r,j}^{s} \end{array}\right.$$

where $f_{i},f_{j}$ represents different GNSS signal frequencies, and the resulting ionosphere-free combination observation equation is as follows:

(23) $$\left\{\begin{array}{l} {\hat{p}}_{IF}=\left\|p_{r}^{n}-{\hat{p}}_{s}^{n}\right\|+t_{r}-t^{s}+m_{\omega }z_{\omega }+\varepsilon_{p,IF}\\ {\hat{L}}_{IF}=\left\|p_{r}^{n}-{\hat{p}}_{s}^{n}\right\|+t_{r}-t^{s}+m_{\omega }z_{\omega }+\lambda_{IF}\cdot N_{IF}+\varepsilon_{L,IF} \end{array}\right.$$

where ${\hat{p}}_{IF},{\hat{L}}_{IF}$ are the noise-inclusive observation values of the pseudorange and carrier phase after ionosphere-free combination; $p_{s}^{n}$ are the satellite coordinates; $t^{s}$ is the receiver clock bias, obtainable from precise orbit and clock data; $z_{\omega },m_{\omega }$ are the tropospheric wet delays and their projection functions, respectively, and the tropospheric delays are usually divided into a kilo component and a wet component, where the kilo component is usually calibrated directly by a priori models, such as the Saastamoinen model [36], while the wet component usually needs to be estimated as a parameter due to its large uncertainty; $p_{r}^{n}$ is the position of the GNSS receiver measurement center in the n-frame; and $\lambda_{IF},N_{IF}$ are the wavelength and integer ambiguity of the ionosphere-free combination. Since the GNSS positioning solution provides the coordinates of the receiver center and the INS mechanical alignment provides the navigation results of the IMU measurement center, which physically do not coincide, a lever arm correction is required during combined navigation solution calculation [37].

(24) $$p_{r}^{n}=p_{b}^{n}+C_{b}^{n}l_{gnss}^{b}$$

where $p_{bi}^{n}$ is the position of the IMU measurement center in the n-frame and $l_{gnss}^{b}$ is the GNSS antenna lever arm. The GNSS factor residual can be expressed as follows:

(25) $$r_{gnss}(z_{i}^{gnss},X)=\left[\begin{array}{l} \left\|p_{b}^{n}+C_{b}^{n}l_{gnss}^{b}-p_{s}^{n}\right\|+t_{r}-t^{s}+m_{\omega }z_{\omega }-{\hat{p}}_{IF}\\ \left\|p_{b}^{n}+C_{b}^{n}l_{gnss}^{b}-p_{s}^{n}\right\|+t_{r}-t^{s}+m_{\omega }z_{\omega }+N_{IF}-{\hat{L}}_{IF} \end{array}\right]$$

### 2.3. Factor Graph Model

**Figure 2:**
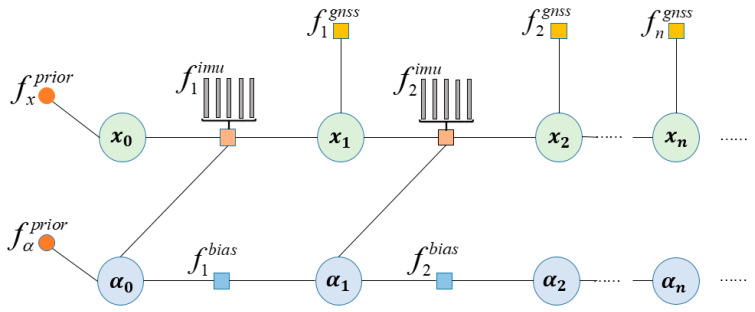
GNSS/INS Combined Positioning Factor Graph Model.

According to the factor graph theory and the previous analysis on factor nodes [38], the factor graph model of combined GNSS/INS positioning can be derived as shown in Figure 2. x is the system state variable and α is the system deviation variable. $f^{prior}$ is the priori factor, which provides the initial estimation for the system state variable and deviation variable. $f^{gnss}$ is the GNSS factor, and $f^{bias}$ is the deviation factor. $f^{imu}$ is the IMU pre-integration factor, which pre-integrates the IMU data with the sampling frequency of GNSS to ensure that the pre-integrated IMU factor and the GNSS factor frequency are consistent. The factor graph combination localization model estimates the state at the initial moment by the a priori factors, while the state variables at the later moment need to be jointly estimated by the state variables and deviation variables at the current moment, and the GNSS factors are also needed to make auxiliary corrections to the state variables.

In the combined GNSS/INS positioning factor graph, the connections between the factor nodes indicate the flow of information. The IMU and GNSS factor nodes are usually independent of each other as they represent different types of sensor measurements, while the state variable nodes are connected to the state variable nodes of the previous moment in order to build a dynamic model of the positioning state. This model is designed to take full advantage of their complementary properties, which can effectively improve the positioning performance and robustness of the positioning system.

## 3. LSTM Neural Network Prediction Assisted Positioning Method

### 3.1. Neural Network Prediction Model Description

**Figure 3:**
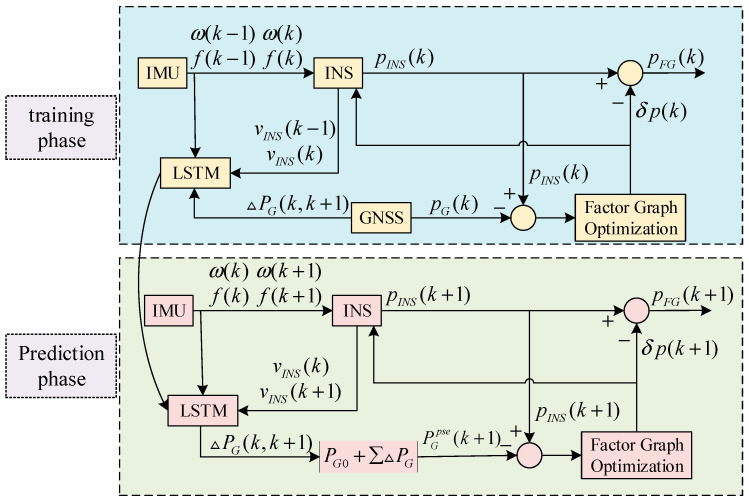
LSTM-Assisted Model Structure Diagram.

In the GNSS/INS integrated systems, numerous neural network-assisted models have already been established. The common idea is to build a relationship between the outputs of the INS (angular velocity, specific force, velocity, position, etc.) and GNSS information. When GNSS signals are available, GNSS information, along with INS outputs, can be used to train a neural network model. In the event of GNSS interruption, pseudo-GNSS information can be obtained from the trained neural network model. Before constructing the neural network model, it is necessary to establish a system model and select appropriate parameters as inputs and outputs to enhance training efficiency and prediction accuracy. Common models include the $O_{INS}-\delta_{GNSS,INS}$ model [39].

(26) $$\begin{array}{c} \delta {\hat{p}}_{GNSS,INS}={\hat{p}}_{\mathrm{GNSS}}-{\hat{p}}_{\mathrm{INS}}\\ \;\;\;\;\;\;\;\;\;\;\;\;=p_{\mathrm{GNSS}}+\delta p_{\mathrm{GNSS}}-(p_{\mathrm{INS}}+\delta p_{\mathrm{INS}})\\ \;\;\;\;\;\;\;\;\;\;\;=\delta p_{GNSS,INS}+\delta p_{\mathrm{GNSS}}-\delta p_{\mathrm{INS}} \end{array}$$

where ${\hat{p}}_{\mathrm{GNSS}},{\hat{p}}_{\mathrm{INS}}$ is the position measurement of the GNSS and INS, respectively; $\delta p_{\mathrm{GNSS}},\delta p_{\mathrm{INS}}$ is the position error of the GNSS and INS, respectively; $p_{\mathrm{GNSS}},p_{\mathrm{INS}}$ is the true value of the position of the GNSS and INS, respectively; and $\delta p_{GNSS,INS}$ is the difference between the position measurement of the INS and GNSS. From Equation (26), it can be seen that the established $O_{INS}-\delta_{GNSS,INS}$ model is always affected by both GNSS and INS errors. To avoid this problem, the $O_{INS}-\Delta P_{GNSS}$ model is proposed.

(27) $$\begin{array}{c} △{\hat{p}}_{GNSS}(k,k+1)={\hat{p}}_{GNSS}(k+1)-{\hat{p}}_{GNSS}(k)\\ \;\;\;\;\;\;\;\;\;\;=p_{GNSS}(k+1)+\delta p_{GNSS}(k+1)-(p_{GNSS}(k)+\delta p_{GNSS}(k))\\ \;\;\;\;\;\;\;\;\;\;=△p_{GNSS}(k,k+1)+\delta p_{GNSS}(k+1)-\delta p_{GNSS}(k) \end{array}$$

where $△{\hat{p}}_{GNSS}(k,k+1)$ is the GNSS position increment in the [k,k+1] time period, and ${\hat{p}}_{GNSS}(k),\delta p_{GNSS}(k)$ is the GNSS position measurement and position error at the k moment, respectively. From Equation (27), it can be seen that the $O_{INS}-△p_{GNSS}$ model is only related to the GNSS position error, avoiding the mixed error of GNSS and INS, which improves the prediction accuracy of the LSTM neural network-assisted model. The specific structure of the LSTM neural network-assisted model is shown in Figure 3.

Where ω represents angular velocity, f represents specific force, $v_{INS},p_{INS}$ represents the velocity and position of the INS, $p_{G}$ is the GNSS position, and δp represents the position error. The LSTM prediction assistance model is divided into two parts: the training phase and the prediction phase. During the training phase, inputs include angular velocity, specific force, and velocity from the previous and current moments of the INS, and the output is the position increment of the GNSS $△p_{G}$. During the training phase, when GNSS signals are strong, the combined positioning accuracy is high and good training data is used to determine the LSTM model parameters in preparation for the prediction phase. During the prediction phase, inputs include the current and next moment’s angular velocity, specific force, and velocity from the INS. Using the LSTM model, the position increment of the GNSS is predicted, and this prediction $p_{G0}$ is added to the initial position information $△p_{G}$ to generate a pseudo-GNSS position $p_{G}^{pse}$, which is used to suppress the divergence of inertial navigation errors.

The accuracy of LSTM training is closely related to network parameters, such as the number of hidden units, learning rate, and optimizer. The accuracy of model training improves with the decrease in the learning rate; however, a too-low learning rate can result in longer training times. A higher number of hidden units can enhance the model’s learning capability but may also lead to overfitting, especially when there is limited training data. The Adam optimizer, utilizing moment estimation, offers advantages such as adaptability, fast computation, and low memory usage, making it a good choice to accelerate training speeds. To mitigate potential overfitting issues, a dropout layer is added after each LSTM layer, enhancing model stability. The final layer of the model is a fully connected layer containing two neurons that output position increments. Specific LSTM parameter settings are shown in Table 1.

### 3.2. LSTM Model

LSTM is a type of recurrent neural network (RNN) [40]. Traditional neural networks or deep neural networks, where nodes within each layer are not interconnected, are suitable for training models that take single sample inputs and produce single sample outputs but are not well-suited for training with sequential data as input. LSTM introduces a complex gating mechanism that can address various shortcomings of traditional and deep neural networks when dealing with sequential data.

**Figure 4:**
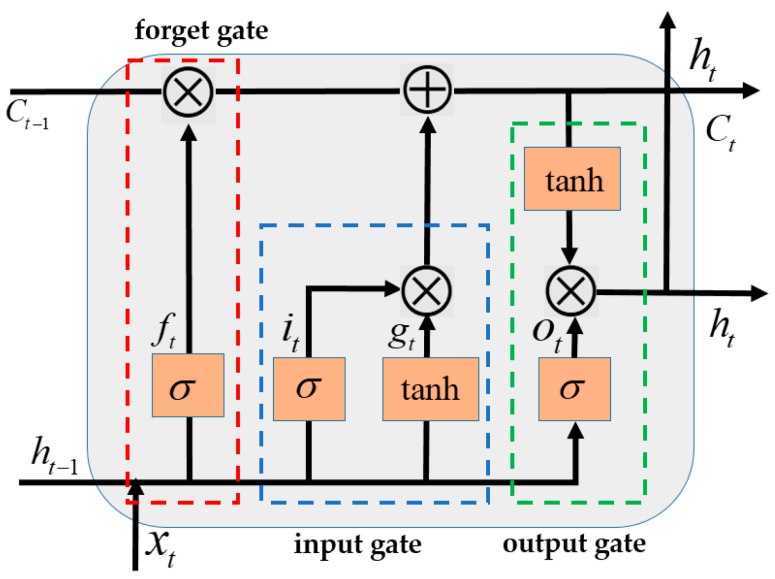
Basic LSTM Structure.

The basic unit of an LSTM includes input gates, forget gates, and output gates [41]. These three main gating structures interact to decide how information is stored, updated, and forgotten, with the basic structure depicted in Figure 4. The forget gate determines which information should be discarded from the cell state, the input gate decides which information from the current input should update the cell state, and the output gate determines which part of the cell state will be used for output. The specific formulas are as follows:

(28) $$f_{t}=\sigma (x_{t}W_{xf}+h_{t-1}W_{hf}+b_{f})$$

(29) $$i_{t}=\sigma (x_{t}W_{xi}+h_{t-1}W_{hi}+b_{i})$$

(30) $$o_{t}=\sigma (x_{t}W_{x0}+h_{t-1}W_{h0}+b_{o})$$

(31) $$c_{t}=f_{t}⊙c_{t-1}+i_{t}⊙g_{t}$$

(32) $$h_{t}=o_{t}⊙\tanh c_{t}$$

Where $f_{t},i_{t},o_{t}$ represents the forget gate, input gate, and output gate, respectively; $x_{t}$ is the input vector; $h_{t}$ represents the hidden unit state; $c_{t}$ is the memory cell state; $g_{t}$ is the candidate memory value for the current time step; ⊙ represents the Hadamard product; $W_{xf},W_{hf},b_{f}$ are the weights and biases of the forget gate; $W_{xi},W_{hi},b_{i}$ are the weights and biases of the input gate; $W_{xo},W_{ho},b_{o}$ are the weights and biases of the output gate; and σ and tanh are activation functions that help avoid problems like exploding or vanishing gradients.

## 4. Experimental Validation and Analysis

### 4.1. Experimental Setup and Data Acquisition

**Figure 5:**
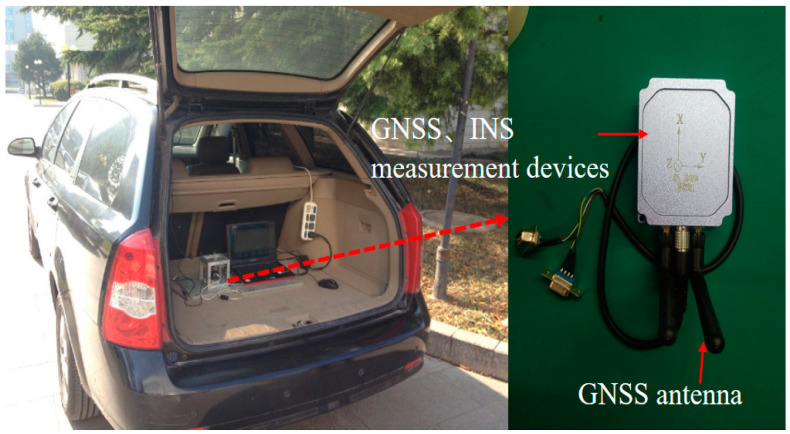
Vehicle with navigation system equipment.

To validate the effectiveness of the proposed LSTM and factor graph neural network-assisted GNSS/INS combined positioning method during GNSS interruptions, road tests were conducted, collecting GNSS and IMU data from a land vehicle platform in an urban environment. The sensors were provided by the Beijing Key Laboratory of High Dynamic Navigation Technology, and the sensor parameters are listed in Table 2. The test data acquisition platform is shown in Figure 5.

**Figure 6:**
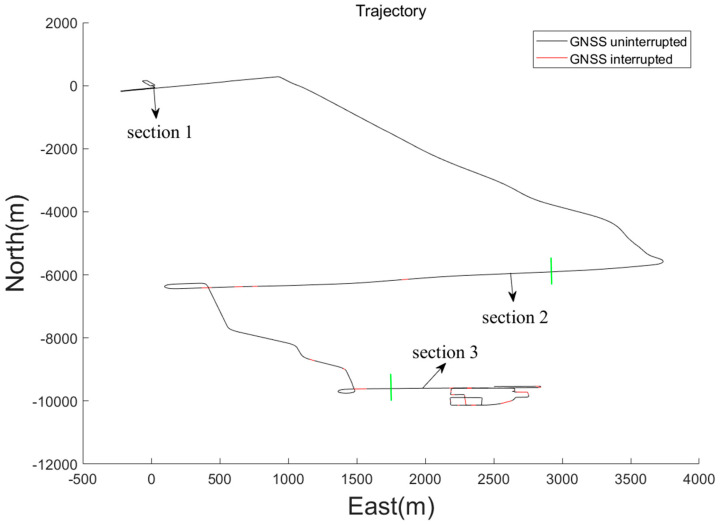
Road Test Trajectory.

The full road test lasted 3270 s, covering a total distance of 21.4 km, with 23 natural interruptions occurring during this period. Based on different testing scenarios, the test trajectory was divided into three sections, separated by green solid lines, as shown in Figure 6. Section 1 lasted 1600 s and was 9.4 km long, primarily on open unobstructed urban highways with no GNSS interruptions, using data from this section to train the LSTM-assisted model and determine LSTM model parameters. Section 2 lasted 850 s and was 7.6 km long, located in urban overpass areas with 7 GNSS interruptions. Section 3 lasted 850 s and was 4.4 km long, mainly in urban areas obstructed by high buildings and structures, with 13 GNSS interruptions. Interruption statistics are shown in Table 3.

### 4.2. Analysis of Experimental Results

**Figure 7:**
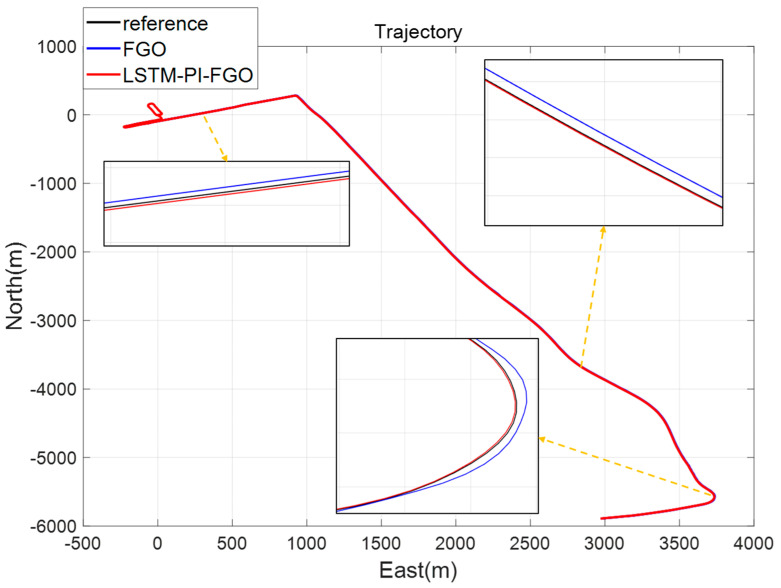
Section 1 Trajectory Comparison.

**Figure 8:**
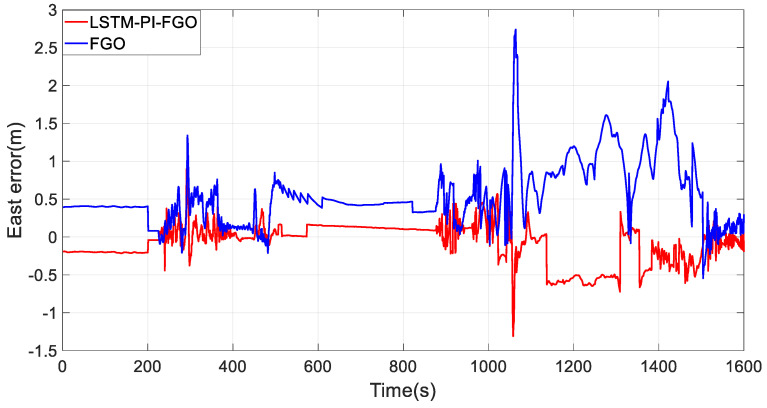
Section 1 East Error.

**Figure 9:**
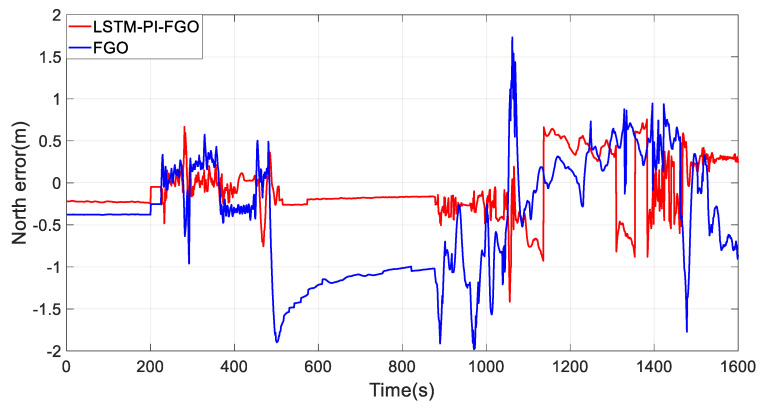
Section 1 North Error.

As depicted in Figure 7, the black line represents the reference trajectory, the blue line represents the factor graph (FGO) calculated trajectory, and the red line represents the LSTM pre-integration factor graph (LSTM-PI-FGO) calculated trajectory. From the trajectory comparison of Section 1, it is evident that in environments with good GNSS, both trajectories based on the FGO combined positioning closely coincide with the reference trajectory, indicating very high positioning accuracy. In conventional FGO, each IMU measurement needs to consider its error model and state transition individually. However, the LSTM-PI-FGO has already considered the state changes and error accumulation during this period, thereby reducing error accumulation. Figure 8 and Figure 9 show that both eastward and northward errors are lower in the LSTM-PI-FGO compared to the FGO.

**Figure 10:**
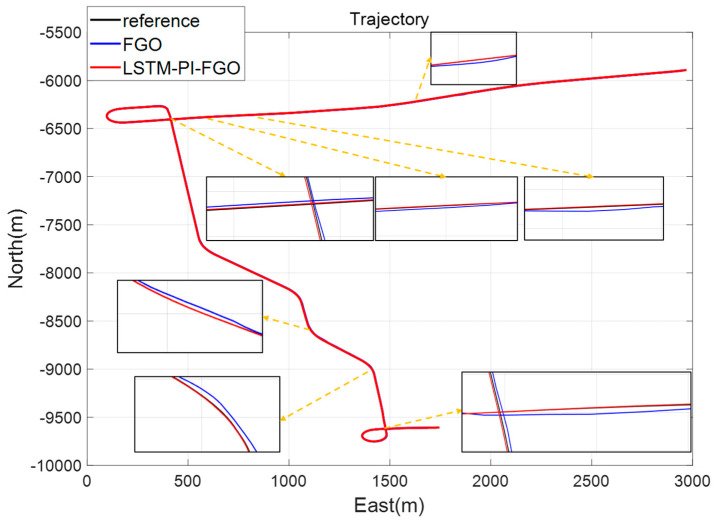
Section 2 Trajectory Comparison.

**Figure 11:**
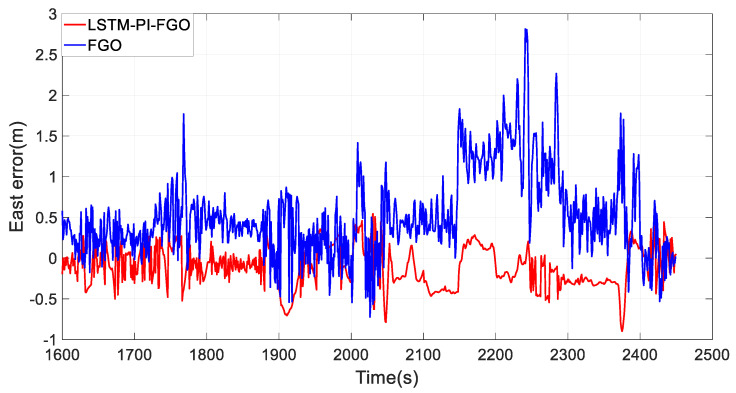
Section 2 East Error.

**Figure 12:**
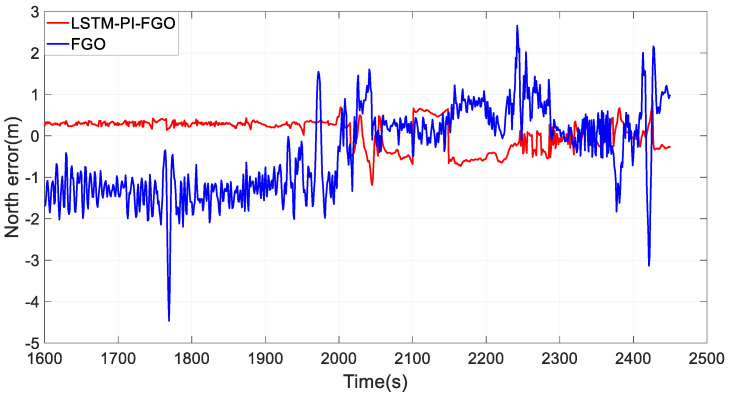
Section 2 North Error.

Section 2, due to overpass obstructions, experienced 7 GNSS interruptions. Combining Figure 10, Figure 11 and Figure 12, after each interruption, the FGO error showed a brief increase before returning to normal, while the LSTM-PI-FGO error showed no significant change. By pre-integrating the IMU, it is possible to effectively isolate IMU errors within each time section, preventing error accumulation throughout the trajectory. This method can reduce the impact of noise in IMU data to some extent, making each time section’s IMU data more independent and reducing long-term dependencies and error propagation. Through LSTM’s GNSS prediction and IMU pre-integration processing, the LSTM-PI-FGO maintained smaller errors throughout Section 2. The experimental results for the road in Section 2 demonstrate that the LSTM-PI-FGO algorithm performs well in the scenario of long straight road sections and is able to provide stable and highly accurate localization services.

**Figure 13:**
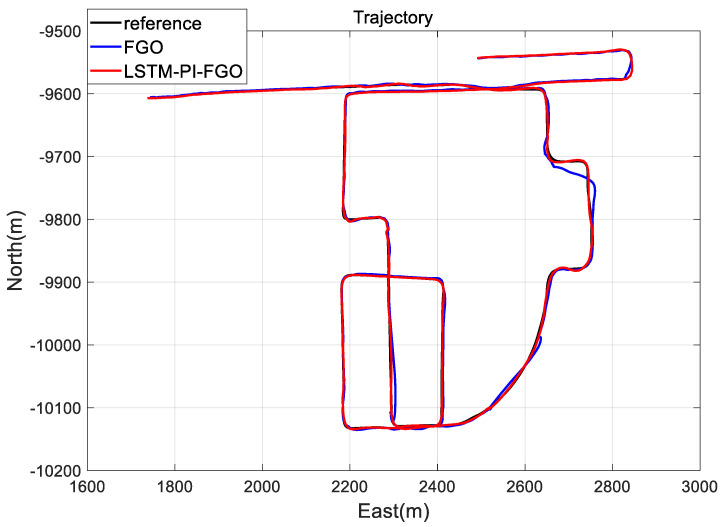
Section 3 Trajectory Comparison.

**Figure 14:**
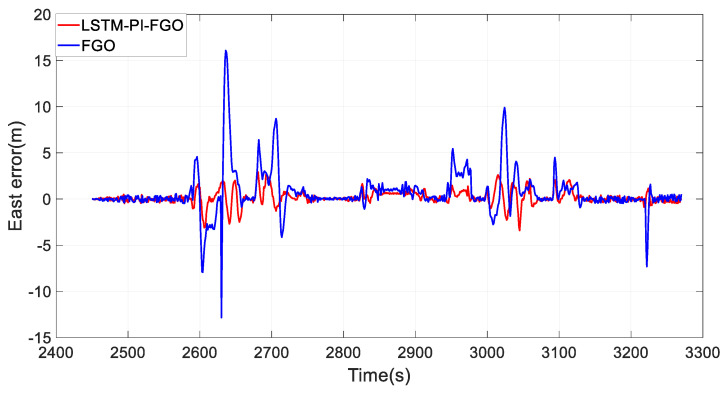
Section 3 Eastward Error.

**Figure 15:**
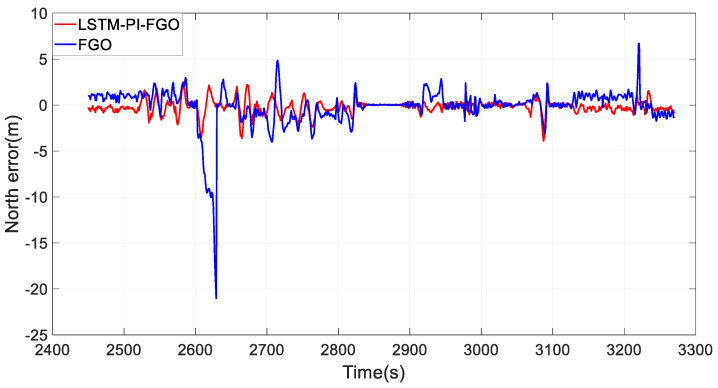
Section 3 Northward Error.

Section 3 experienced frequent interruptions, including curve interruptions and other complex situations, with trajectory comparisons shown in Figure 13 and eastward and northward error curves shown in Figure 14 and Figure 15. During GNSS interruptions, the LSTM-PI-FGO, by predicting GNSS position increments and generating pseudo-GNSS information for factor graph fusion, enhanced the accuracy of combined positioning. Compared to the FGO algorithm, the root mean square errors for eastward and northward directions were reduced from 2.40 m and 2.16 m to 0.79 m and 0.79 m, respectively, while the maximum errors were reduced from 16.08 m and 21.02 m to 3.41 m and 3.89 m. It is evident that in environments with frequent GNSS interruptions, through LSTM’s predictions, the maximum positioning errors in the eastward and northward directions were significantly reduced, ensuring the robustness of the combined positioning system.

The error statistics of road sections 1, 2, and 3 are shown in Table 4. In Section 1, with good GNSS signal, by pre-integrating the FGO, the root mean square error of LSTM-PI-FGO position is reduced by 58.7%, and the maximum error is reduced by 42.9%; in Section 2, with short interruptions of the GNSS signal, by predicting the GNSS position information from the LSTM, the root mean square error of LSTM-PI-FGO position is reduced by 62.2%, and the maximum error is reduced by 71.7%; in Section 3, where GNSS signals are frequently and briefly interrupted, the LSTM-PI-FGO position root mean square error is reduced by 65.3% and the maximum error is reduced by 80.5% by predicting the GNSS position information from the LSTM.

## 5. Conclusions

In complex urban areas, it is inevitable to encounter short-term GNSS interruptions, resulting in reduced accuracy of combined GNSS/INS positioning. In this paper, we propose an LSTM-assisted GNSS/INS pre-integrated factor graph combined positioning method, which combines the predictive capability of LSTM and the data fusion of the factor graph technique in order to achieve high-precision positioning when GNSS signals are unavailable. By comparing the experiments with the factor graph, the experimental results show that the LSTM model can effectively utilize the historical GNSS and INS data to predict the GNSS position during the interruption period, and all the error indexes of the combined positioning are significantly reduced. Through this method, the challenges brought by GNSS interruptions can be effectively solved. The LSTM-assisted GNSS/INS pre-integrated factor graph combined localization method effectively combines the advantages of deep learning and traditional navigation and positioning algorithms and provides a new solution to deal with the problem of GNSS signal interruptions. However, further research on the selection and matching of the model of neural network and the optimization of the factor graph is still needed. Meanwhile, more sensors will be considered to be fused in the future, and the factor graph-based combined positioning will be applied to different real-world scenarios.

## Figures and Tables

**Table 1:** Network model parameters.

Parameter Name	Value
Learning Rate	0.005
Learning Rate Decay Factor	0.5
Number of Hidden Units	100
Number of Epochs	200
Optimizer	Adam

**Table 2:** Sensor Parameters.

Sensor	Parameters	Accuracy
IMU	Gyroscope Bias	20°/hr
	Gyroscope Random Walk	$0.0667{}^{\circ}/\sqrt{\mathrm{hr}}$
	Accelerometer Bias	50 mg
	Sampling Frequency	100 Hz
GNSS	Position Accuracy	2 m
	Sampling Frequency	1 Hz

**Table 3:** GNSS Interruption Time Statistics.

Section	Start Time (s)	Interruption Duration (s)	Section	Start Time (s)	Interruption Duration (s)
2	1765	6	3	2696	17
2	1928	5	3	2758	9
2	1944	7	3	2813	2
2	1968	7	3	2897	2
2	2240	5	3	2985	5
2	2283	4	3	3002	27
2	2416	9	3	3038	6
3	2525	4	3	3085	6
3	2545	6	3	3109	6
3	2598	3	3	3147	4
3	2612	15	3	3215	8
3	2628	2	3	3231	4
3	2635	8			

**Table 4:** Positioning Error Statistics.

Section	Algorithm	Orientation	RMSE (m)	Maximum Error (m)
1	LSTM-PI-FGO	east	0.27	1.31
		north	0.33	1.41
	FGO	east	0.69	2.74
		north	0.78	1.98
2	LSTM-PI-FGO	east	0.27	0.90
		north	0.37	1.19
	FGO	east	0.73	2.81
		north	1.10	4.46
3	LSTM-PI-FGO	east	0.79	3.41
		north	0.79	3.89
	FGO	east	2.40	16.08
		north	2.16	21.02

## Data Availability

The data presented in this study are available on request from the corresponding author.
